# Evaluation of the Cell Behavior and Growth Characteristics of the Porcine Dermal Xenograft Patch in Relation to the Surface Properties

**DOI:** 10.3389/fbioe.2022.811446

**Published:** 2022-05-30

**Authors:** Duygu Aydemir, Ilker Eren, Mehmet Demirhan, Nuriye Nuray Ulusu

**Affiliations:** ^1^ Department of Medical Biochemistry, School of Medicine, Koc University, Istanbul, Turkey; ^2^ Koc University Research Center for Translational Medicine (KUTTAM), Istanbul, Turkey; ^3^ Department of Orthopedic Surgery, School of Medicine, Koc University, Istanbul, Turkey

**Keywords:** oxidative stress, surface properties, dermal xenograft patch, trace element, mineral

## 1 Introduction

**FIGURE 1:**
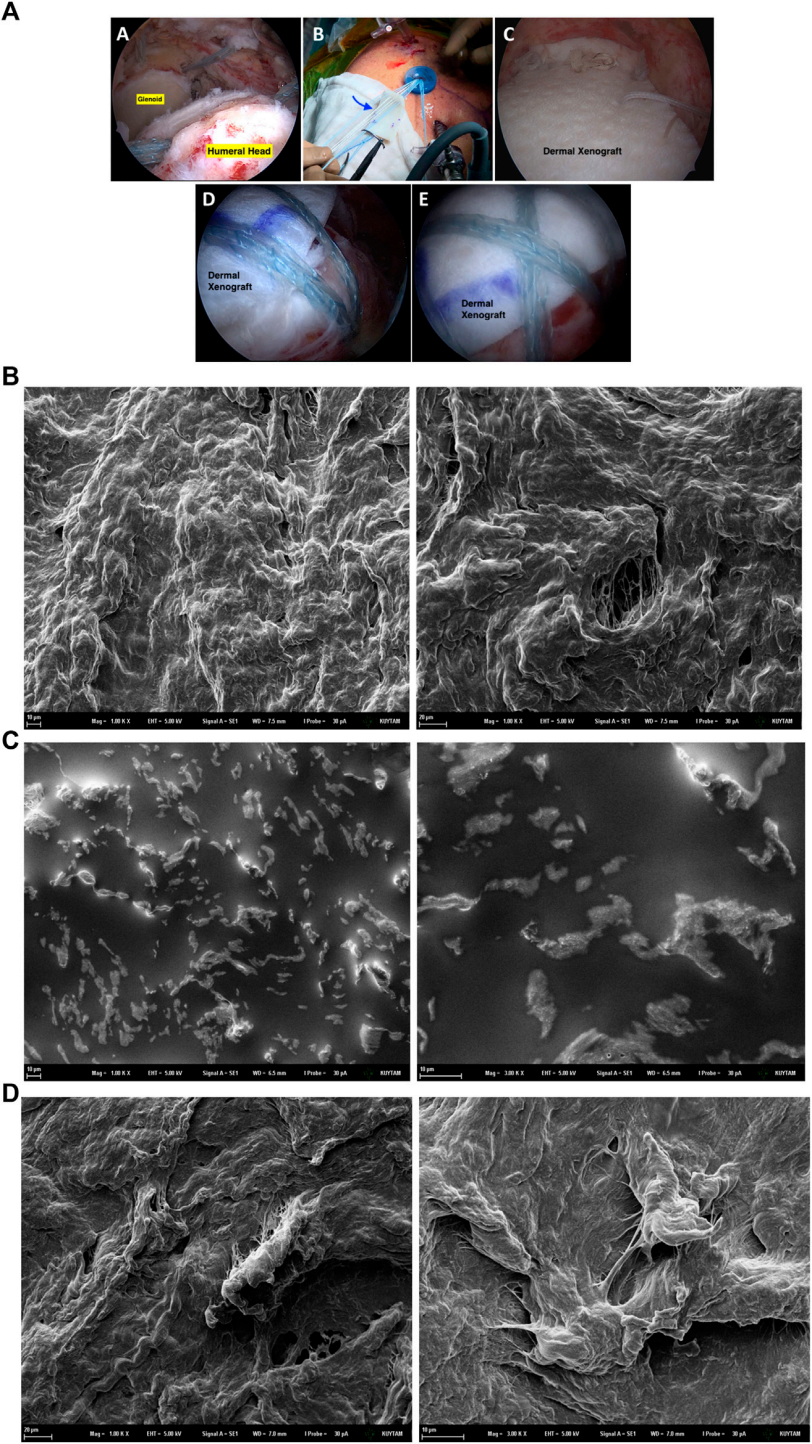
A sample of surgical use of dermal xenograft as tendon substitute **(A)**. In irreparable rotator cuff tears of the shoulder **(A)**, superior capsular reconstruction is one of the popular surgical options. Immediately after the graft is shaped according to the defect size, it is introduced to the joint using shuttle sutures via arthroscopy portal **(B)**. After appropriate fixation with anchors, graft acts as the superior capsule, preventing superior migration **(C,D,E)**. SEM images of the untreated xenograft dermal patch **(B)**, xenograft dermal patch incubated in the DMEM cell media for 48 h at 37°C in the incubator **(C)** and Xenograft dermal patch incubated with 293T epithelial cells in the DMEM cell media for 48 h at 37°C in the incubator **(D)**.

The rotator cuff (RC) plays a significant role in the glenohumeral joint function as a dynamic stabilizer of the joint since RC allows a full range of movement and provides stability. This demanding function renders RC tendons an ideal candidate for degenerative problems. The prevalence of RC tears in the general population is 34% and even higher in the elderly population exceeding 50% (Sher et al., 1995; Tempelhof et al., 1999). RC tears enlarge over time because RC muscles undergo atrophy and fatty degeneration (Yamaguchi et al., 2001). Surgical options for RC tears include debridement to latissimus dorsi tendon transfer and reverse total shoulder arthroplasty. When untreated, the natural course of disease eventually results in irreparable massive RC tears with irreversible muscle changes. On the other hand, RC tears are too complex to be treated with simple methods, and a patient can be too young to sacrifice the joint with arthroplasty (Ono et al., 2016). Therefore, indicating specific patient groups has become one of the most prevalent dilemmas and difficulties for shoulder surgeons in the last decades. Several authors proposed a novel but non-anatomic reconstructive approach to this old problem: reconstruction of the superior capsule (SCR). The method comprised of re-creating the lost glenohumeral dynamic stabilizer (rotator cuff) with a static restraint (the superior joint capsule) (Figure 1A) (Badhe et al., 2008; Mihata et al., 2013; Burkhart et al., 2016; Boutsiadis et al., 2017; Denard et al., 2018; Yildiz et al., 2019). The method was first proposed using tensor fascia lata (TFL) autograft, and time-zero mechanical success was proven in cadaveric studies (Mihata et al., 2012, 2013).

Orthobiologics have been used in orthopedic surgery that is expected to have high biocompatibility and induce cell proliferation by enabling cells to attach to their surfaces (Toolan, 2006; Aurora et al., 2012; Mihata et al., 2012; Zhao et al., 2017; Bravo et al., 2018). Due to the donor site morbidity, acellular dermal matrix grafts (xenografts, allografts, autografts) and synthetic grafts were proposed as an alternative option. Fascia lata femoris is used as an autograft to care for RC tears by harvesting a band of fascia. Allografts are obtained from tendon tissues of human dermal origin; however, residual cells should be removed to avoid immune responses. On the other hand, allografts obtained depend on donors’ availability and tissue properties. Synthetic grafts can be categorized as degradable and non-degradable with superior strength, consistency, and durability (Ono et al., 2016). The xenografts are porcine-derived acellular dermal matrix scaffolds and were popularized as a low-cost alternative to the human-derived allografts (Barber, 2018; Flury et al., 2018; Kalina et al., 2019; Polacek, 2019; Ravenscroft et al., 2019). Today, the superiority of dermal xenografts is still debated because they are cheaper and easier to obtain. On the other hand, dermal allografts are more reliable from a biological perspective, such as low complication rates, enhanced biocompatibility, and lower immune response risk (Yildiz et al., 2019).

Although all these options restore time-zero acromial contact pressures and restrain superior translation, grafts from all sources should heal to supraglenoid tubercule of glenoid and major tubercule of the humerus to achieve good results in the long term. Although human-derived dermal allografts and fascia lata allografts have comparable outcomes, porcine-derived dermal xenograft has less favorable and inconstant results in the literature. Despite similar time-zero characteristics, less promising results in the follow-up were attributed to inferior biological response and failure of healing in the bone-graft interface (Ferrando et al., 2021). The reasons for the failure of these materials need detailed specific research on the cellular level since various enzymatic reactions and molecular signaling factors involve wound healing and cell proliferation. Moreover, there are different types of wounds, and every kind of wound has different healing requirements.^22-25^ Scaffolding materials, including xenografts and allografts, have specific characteristics; therefore, they induce different molecular and cellular signaling mechanisms in wound healing which are still poorly understood (Mir et al., 2018; Chouhan and Mandal, 2020). Additionally, xenografts have been reported as inferior to other acellular dermal matrixes (Ono et al., 2016). On the other hand, a novel basement membrane epithelium on the tendon tissue has been reported that is required for cell retention and fibrous adhesions formed following RF tears (Taylor et al., 2011; Kalem, 2015). For the first time in the literature, we investigated the ability of epithelial cell growth on the xenograft patch correlated with surface properties, biochemical properties, trace elements, and mineral metabolism.

Therefore, in this study, we have utilized 6 × 8 cm decellularized porcine dermal xenograft (Arthrex DX Reinforcement Matrix, Naples, FL, United States) to evaluate the behavior and growth characteristics of epithelial cells on the xenograft patch in relation to surface characteristics. The manufacturing process of the patch consists of harvesting, disinfection, viral inactivation, and cell removal processes while preserving the open pore structure of the collagen matrix. We have investigated the usage of trace elements and minerals by the cells, oxidative stress metabolism, and positions of the cells growing on the patch to show the cellular requirements of cells when they are growing. Our data may help to reveal possible mechanisms and conditions of cells growing on the xenograft patches, which may be used to improve patch properties in the future.

## 2 Materials and Methods

### 2.1 Materials

Porcine dermal xenograft was purchased from Arthrex DX Reinforcement Matrix (United States ). 293T cells were obtained from ATCC (United Kingdom). 65% nitric acid (HNO_3_) SUPRAPUR^®^ grade was purchased from Merck (United States ). DMEM, phosphate buffer saline (PBS), fetal bovine serum (FSB), and penicillin-streptomycin were obtained from GIBCO (United Kingdom). All other chemicals were purchased from Sigma-Aldrich (United States ).

### 2.2 Cell Culture

Experiments were conducted with human embryonic kidney epithelial cells (293T) cultured in a DMEM growth medium, supplemented with 10% heat-inactivated fetal bovine serum (FBS) and 1% pen-strep. Cells were kept in the incubator at 37°C under 5% CO_2_. Firstly, cells were expanded in the 75 cm^2^ flasks until they reached 80–90% confluency. Cell growth and characteristics were observed every 24. hour under the light microscope. Cells were then collected from 75 cm^2^ flasks via trypsin-EDTA (0.05%) and centrifuged at 2000 rpm for 3 min. The cell pellet was suspended in the indicated DMEM media and seeded into 6-well plates containing sterile xenograft patch, and cells seeded into plates without xenograft patch were used as control groups.

#### 2.2.1 Preparation of cell lysates for evaluation of enzyme activities

293T cells were seeded on the xenograft patch with DMEM supplemented with 10% FBS and 1% pen-strep. After 48 h incubation, xenograft patches were replaced into the new Petri dishes, and 1–2 ml of trypsin-EDTA was added onto the patches for incubation at 37°C for 2–3 min. Afterward, detaching cells from patches were collected in DMEM media, and samples were centrifuged at 2000 rpm for 2 min at 4°C. After centrifugation, the supernatant was removed, and the cell pellet was suspended in the sodium phosphate buffer (pH 7.4) containing protease inhibitor cocktail and sonicated for 10 s on the ice. Afterward, samples were centrifuged at 33.800 rpm for 1 h at 4°C in the ultracentrifuge and stored at -80°C until experiments were performed.

### 2.3 Fourier Transform Infrared (FTIR) Spectroscopy

FTIR was performed to compare untreated xenograft with cell culture treatment-applied ones. Dermal xenograft patches were treated with cell media alone and with 293T cells for 48 h at the 37° incubator. The FTIR analysis was performed using Thermo-Scientific iS10 FTIR equipment. Each sample was scanned 64 times with 4 cm-1 resolution at room temperature and atmospheric pressure. The data were collected in 4,000–650 cm^−1^ spectral windows.

### 2.4 Scanning Electron Microscope (SEM) Analysis

Xenograft samples were analyzed after 48 h of incubation with cell media alone and 293T cells, respectively, to evaluate whether there was any change in the surface topography. Samples were coated with a 15-nm-thick gold layer, and Zeiss ultra plus field emission scanning electron microscope (SEM) was used for these observations. A secondary electron detector was used to obtain micrographs. 10 kV acceleration voltage and 8.1 mm working distance was used.

### 2.5 Inductively Coupled Plasma Mass Spectrometry (ICP-MS)

Cell media samples were collected from plates in which cells alone and xenograft patch, respectively. DMEM media supplemented with 10% FBS, and 1% pen-strep was used to compare cell nutrient consumption of the cells growing on the xenograft patch. After 48 h, cell media was collected from flasks and diluted as 1/10 in the 65% SUPRAPUR® HNO_3_. Trace and mineral elements in rat tissue and serum samples were measured by Agilent 7700xICP-MS (Agilent Technologies Inc., Tokyo, Japan). Helium gas is used in the collision/reaction cell to remove potential polyatomic interferences. MassHunter Work Station software is used for the data analysis. The analysis mode “spectrum” was chosen for each sample; the peak pattern was drawn from three points (Aydemir et al., 2020b)**.**

### 2.6 Protein Determination in the Cell Lysates

Protein concentrations in cell lysates were evaluated via the BCA method according to the kit instructions of Pierce™ BCA Protein Assay Kit (Pierce™ BCA Protein Assay), and albumin was used to prepare the standard curve.

### 2.7 Evaluation of Enzyme Activities Related to Cell Proliferation

0.2 mM NADP+, 10 mM MgCl_2_, and 0.6 mM glucose-6 phosphate (G6P) in 100 mM Tris-HCl buffer (pH 8.0) were used to evaluate glucose 6-phosphate dehydrogenase activity (G6PD) in the cells grown on the patch. Enzyme activity was measured by Synergy H1 BIOTEK spectrophotometer production at 340 nm at 37°C for 60 s 6-phosphogluconate dehydrogenase (6-PGD) activity was measured the same as G6PD activity; only 0.6 mM 6-phosphogluconate (6-PG) was used instead of (G6P) (Aydemir et al., 2020a).

### 2.8 Evaluation of Oxidative Stress Metabolism of Cells

A reaction mixture with 1 mM oxidized glutathione (GSSG), 100 mM sodium phosphate buffer (pH 7.4), and 0.2 mM NADPH was used to measure glutathione reductase (GR) activity in the cells grown on the dermal patch. Glutathione s-transferase (GST) enzyme activity was calculated by the conjugation of GSH with 1-chloro-2,4-dinitrobenzene (CDNB). The incubation mixture has contained 0.2 M sodium phosphate buffer (pH 6.5), 25 μL of 20 mM GSH, 25 μL of 20 mM CDNB. The reaction was followed for 60 s at 340 nm for GR and GST enzyme measurements (Aydemir et al., 2020a).

### 2.9 Statistical Analysis

Statistical analysis of data was performed by GraphPad Prism software. All data were analyzed either by one-way or two-way analysis of variance (ANOVA) followed by a Tukey post hoc test for multiple comparisons. The results are represented as the mean ± standard deviation (SD) of three independent experiments. *p* ≤ 0.05 was considered significant.

## 3 Results

### 3.1 Influence of the cell culture environment on the surface properties of the porcine dermal patch

The influence of the cell media alone and cell growth on the surface properties of the dermal patch was investigated via SEM analysis. The porous structure of the dermal patch has been observed under SEM microscopy (Figure 1B). After 48 h of incubation within the cell media, the porous surface of the patch was flattened compared to the untreated patch. Additionally, potassium chloride (KCl) and sodium chloride (NaCl) salts have been observed on the surface as bright regions (Figure 1C) as we described previously as well (Aydemir et al., 2021)**.** The patch was incubated with 293T cells for 48 h; however, we did not observe cell clusters attached to the patch; controversially, a few cells could attach and grow on the patch after 48 h (Figure 1D).

### 3.2 Evaluation of the Proliferation of Cells Growing on the Dermal Patch

**FIGURE 2:**
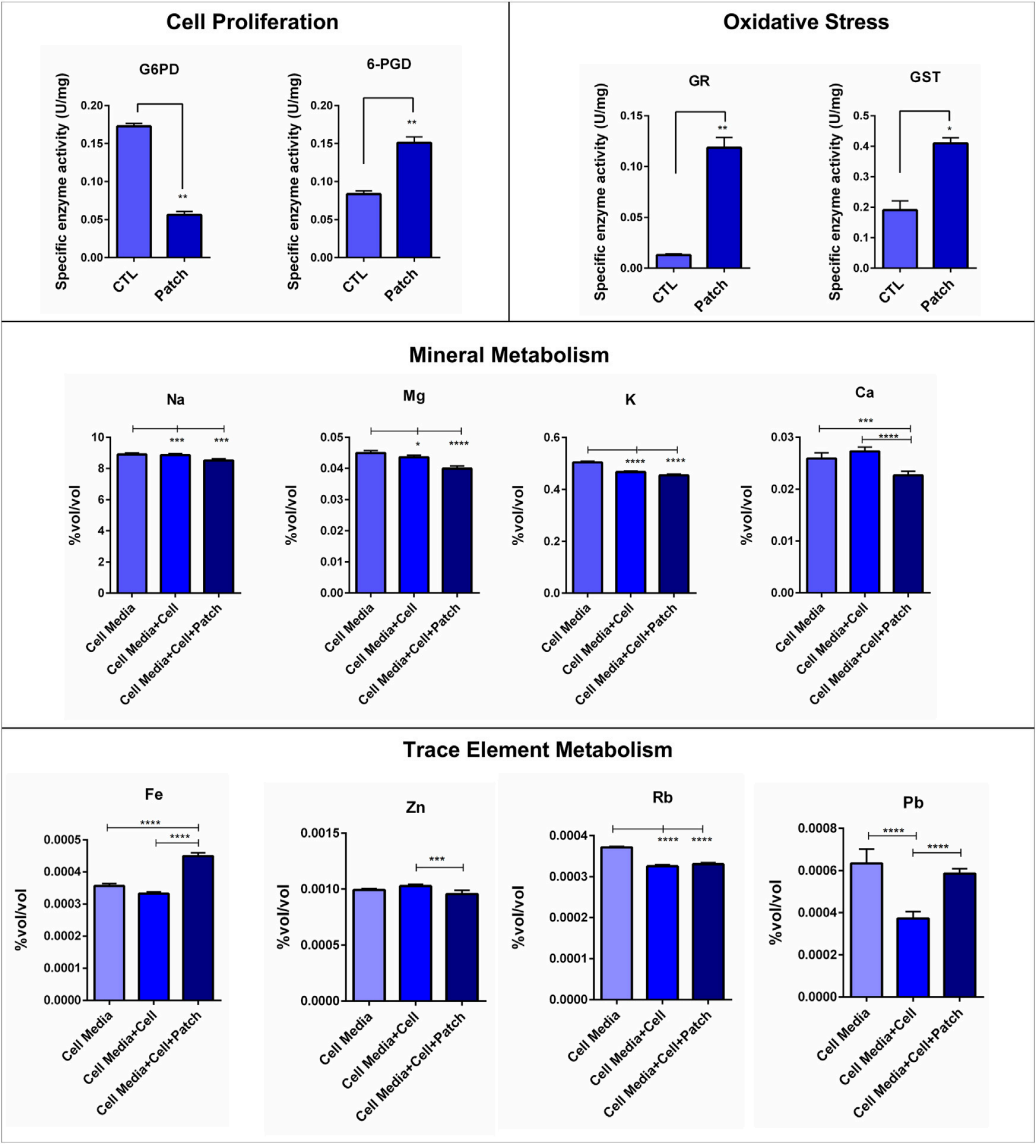
Evaluation of the proliferation and oxidative stress status of cells growing on the dermal patch via G6PD, 6PGD, GR and GST enzyme activities involving in the pentose phosphate pathway. Na, Mg, K, Ca, Fe, Cu, Sr, Zn, Rb and Pb levels in the cell media of the dermal patch with and without cells were evaluated via ICP-MS. Notes: *p < 0.05, **p < 0.001 and ***p < 0.0001.

G6PD and 6-PGD enzyme activities in the cells growing on the dermal patch and Petri dish were a measure to evaluate the cell proliferation status of the cells. G6PD activity significantly decreased in the cells growing on the dermal patch compared to the control cells, which are growing on the Petri dish; controversially, 6-PGD enzyme activities increased dramatically in the cells growing on the patch (Figure 2).

### 3.3 Oxidative Stress Enzymes Significantly Increased in the Cells Growing on the Patch

Anti-oxidant enzymes, including GR and GST, were measured to evaluate the oxidative stress levels of the cells. GR and GST enzymes were significantly increased in the cells growing on the dermal patch compared to the cells growing on the cell culture flask (Figure 2).

### 3.4 The Xenograft Patch did Not Affect the Mineral Balance of the Cells

Na, Mg, K, and Ca levels in the cell media alone, cell media within 293T cells grown, and cell media within 293T cells grown on the dermal patch were measured via ICP-MS. Na, Mg, K, and Ca levels did not significantly change between cells grown on the Petri dish and dermal patch (Figure 2).

### 3.5 Trace Metabolism of the Cells Was Affected by the Dermal Patch

Cu and Sr levels did not change between the groups. Zn and Rb levels did not change between the cells growing on the flask and cells growing on the dermal patch. Fe levels significantly increased in the cells growing on the dermal patch compared to the other groups. Moreover, Cs, Pb, Ti, and Ni levels significantly increased in the cells growing on the dermal patch compared to those growing on the plate (Figure 2).

## 4 Discussion

Firstly, we aimed to investigate the influence of the cell culture environment and cell growth on the dermal patch. Therefore, the dermal patch was incubated within cell media and cell media with cells for 48 h, respectively. SEM analysis showed that the surface of the dermal patch was flattened when set within cell media, and salt particles were formed on the patch (Figures 1B–D), as we reported previously (Aydemir et al., 2021). On the other hand, cells could not attach and grow as clusters on the dermal patch, proven by the SEM pictures (Figure 1D). This undesirable event can result from the flattening of the dermal patch in the cell culture conditions; however, a successful dermal patch should have high porosity to increase the surface area for the cell attachment, cell proliferation, homogenous distribution of the cells on the patch and effective transport of minerals and nutrients (Baum and Arpey, 2006; Bainbridge, 2013). On the other hand, we have performed FTIR analysis; however, there was no significant difference between xenograft patches seeded with cells and without cells (Supplementary Material S1).

Orthobiologics are used to enhance wound healing, consisting of different stages as the inflammatory phase, proliferative phase, and remodeling phase. Dermal xenograft patches after inflammatory responses have been investigated, but proliferative and remodeling properties of a dermal patch vary depending on the wound and host response. Since our study design is based on the proliferative phase of the disease, we have started our experiments by investigating cell adhesion and proliferation to dermal patch in relation to surface properties of patch modified by cell culture environment (Clark, 1993; Greenhalgh, 1998; Zarei and Soleimaninejad, 2018).

We investigated G6PD and 6-PGD enzymes to evaluate the cell proliferation of the cells growing on the dermal patch. G6PD is the rate-limiting step of the pentose phosphate pathway (PPP) responsible for immune response, cell growth, and proliferation (Aydemir et al., 2019a; 2019b)^.^ G6PD levels significantly decreased, almost three times in the cells growing compared to those expanded on the Petri dish (Figure 2). However, 6-PDG levels increased dramatically in the cells growing on the dermal patch (Figure 2). 6-PGD involves in the PPP and regulates several pathways of non-specific immune responses. PPP is known as the gatekeeper of inflammation by involving in the glutathione metabolism via the production of NADPH (Spolarics and Navarro, 1994; Xue and Jackson, 2015; Perl, 2017).

GR and GST are the major enzymes that regulate glutathione metabolism by protecting the GSH/GSSG ratio. NADPH is produced by G6PD and 6-PGD enzymes and used by GR to convert GSSG into GSH, the desired form of glutathione (Aydemir and Ulusu, 2020; Aydemir et al., 2020a). Our data showed that GR and GST enzyme levels significantly increased in the 293T cells growing on the dermal patch compared to the cells expanded on the Petri dish, which shows increased oxidative stress levels (Figure 2). High levels of the 6-PGD can be associated with increased levels of oxidative stress and possible inflammatory response (Spolarics and Navarro, 1994; Xue and Jackson, 2015; Perl, 2017). Increased levels of oxidative stress have adverse effects on inflammatory responses and cell proliferation and can be a biomarker for biocompatibility (Bernard et al., 2018). Since surface properties of the dermal patch were not perfectly suitable for cell attachment and growth, few cells attached to the patch, and decreased cell proliferation and enhanced oxidative stress have been observed (Figure 2).

On the other hand, orthobiologics should enable cells to distribute homogeneously on the patch and supply effective nutrient transport (Spolarics and Navarro, 1994; Nauta et al., 2011). Mineral levels including Na, Ca, Mg, and K in the cell media of each group were measured to evaluate the mineral metabolism of the cells (Aydemir et al., 2019a; 2020b; 2020a, 2021). Our data showed that indicated mineral levels in the cell media in which cells expanded did not significantly change compared to the cell media within cells growing on the dermal patch (Figure 2). There was only a slight decrease in the cells growing on the patch, but that may have resulted from the formation of mineral salts on the patch observed via SEM (Figure 2B), as we have reported previously on PDMS biomaterials as well (Aydemir et al., 2021)**.** Moreover, we investigated trace element levels in the cells. Zn levels significantly increased in the cells growing on the patch compared to the other groups, which may result from increased levels of oxidative stress, as we reported previously. Fe is required for cell cycle progression and cell proliferation; our data showed cells growing on the patch consumed less Fe compared to the control cells (Figure 2). (Aydemir et al., 2019a; 2020b; 2020a, 2021).

In conclusion, dermal xenografts and allografts are widely used orthobiologics to treat RC tears or reconstruct glenohumeral joint biomechanics. However, the advantages of dermal patches are still debated. Our data showed that dermal porcine xenograft did not effectively support cell adhesion and proliferation, resulting in a decrease in the surface porosity and losing the integrity of the dermal patch. Thus, the xenograft dermal patch may not suit this clinical setting. Utilizing autologous tissue instead of xeno- or allograft may provide reliable healing from a biological perspective.

## Data Availability

The original contributions presented in the study are included in the article/Supplementary Material, further inquiries can be directed to the corresponding author.

## References

[B1] Aurora A. McCarron J. A. van den Bogert A. J. Gatica J. E. Iannotti J. P. Derwin K. A. (2012). The Biomechanical Role of Scaffolds in Augmented Rotator Cuff Tendon Repairs. J. Shoulder Elb. Surg. 21, 1064–1071. 10.1016/j.jse.2011.05.014 21885301

[B2] Aydemir D. Dogru S. Alaca B. E. Ulusu N. N. (2021). Impact of the Surface Modifications and Cell Culture Techniques on the Biomechanical Properties of PDMS in Relation to Cell Growth Behavior. Int. J. Polym. Mater. Polym. Biomaterials, 1–12. 10.1080/00914037.2021.1919670

[B3] Aydemir D. Hashemkhani M. Acar H. Y. Ulusu N. N. (2020a). Evaluation of the Biocompatibility of the GSH-Coated Ag2S Quantum Dots *In Vitro*: a Perfect Example for the Non-toxic Optical Probes. Mol. Biol. Rep. 47, 4117–4129. 10.1007/s11033-020-05522-3 32436042

[B4] Aydemir D. Hashemkhani M. Acar H. Y. Ulusu N. N. (2019a). *In Vitro* interaction of Glutathione S‐transferase‐pi Enzyme with Glutathione‐coated Silver Sulfide Quantum Dots: A Novel Method for Biodetection of Glutathione S‐transferase Enzyme. Chem. Biol. Drug Des. 94, 2094–2102. 10.1111/cbdd.13614 31452310

[B5] Aydemir D. Hashemkhani M. Durmusoglu E. G. Acar H. Y. Ulusu N. N. (2019b). A New Substrate for Glutathione Reductase: Glutathione Coated Ag2S Quantum Dots. Talanta 194, 501–506. 10.1016/j.talanta.2018.10.049 30609564

[B6] Aydemir D. Simsek G. Ulusu N. N. (2020b). Dataset of the Analyzing Trace Elements and Minerals via ICP-MS: Method Validation for the Mammalian Tissue and Serum Samples. Data Brief 29, 105218. 10.1016/j.dib.2020.105218 32071990 PMC7016227

[B7] Aydemir D. Ulusu N. N. (2020). Comment on the: Molecular Mechanism of CAT and SOD Activity Change under MPA-CdTe Quantum Dots Induced Oxidative Stress in the Mouse Primary Hepatocytes (Spectrochim Acta A Mol Biomol Spectrosc. 2019 Sep 5; 220:117104). Spectrochimica Acta Part A Mol. Biomol. Spectrosc. 229, 117792. 10.1016/j.saa.2019.117792 31865110

[B8] Badhe S. P. Lawrence T. M. Smith F. D. Lunn P. G. (2008). An Assessment of Porcine Dermal Xenograft as an Augmentation Graft in the Treatment of Extensive Rotator Cuff Tears. J. Shoulder Elb. Surg. 17, S35–S39. 10.1016/j.jse.2007.08.005 18201655

[B9] Bainbridge P. (2013). Wound Healing and the Role of Fibroblasts. J. Wound Care 22, 407–412. 10.12968/jowc.2013.22.8.407 23924840

[B10] Barber F. A. (2018). Editorial Commentary: Don't Pig Out when Selecting a Shoulder, Rotator Cuff Augmentation Graft! Xenografts Are Not the Way to Go. Arthrosc. J. Arthrosc. Relat. Surg. 34, 38–40. 10.1016/j.arthro.2017.07.013 29304975

[B11] Baum C. L. Arpey C. J. (2006). Normal Cutaneous Wound Healing: Clinical Correlation with Cellular and Molecular Events. Dermatol. Surg. 31, 674–686. 10.1111/j.1524-4725.2005.31612 15996419

[B12] Bernard M. Jubeli E. Pungente M. D. Yagoubi N. (2018). Biocompatibility of Polymer-Based Biomaterials and Medical Devices - Regulations,in Vitroscreening and Risk-Management. Biomater. Sci. 6, 2025–2053. 10.1039/C8BM00518D 29968869

[B13] Boutsiadis A. Chen S. Jiang C. Lenoir H. Delsol P. Barth J. (2017). Long Head of the Biceps as a Suitable Available Local Tissue Autograft for Superior Capsular Reconstruction: "The Chinese Way". Arthrosc. Tech. 6, e1559–e1566. 10.1016/j.eats.2017.06.030 29354474 PMC5709836

[B14] Bravo D. Jazrawi L. Cardone D. A. Virk M. Passias P. G. Einhorn T. A. (2018). Orthobiologics A Comprehensive Review of the Current Evidence and Use in Orthopedic Subspecialties. Bull. Hosp. Jt. Dis. (2013) 76, 223–231. 31513506

[B15] Burkhart S. S. Denard P. J. Adams C. R. Brady P. C. Hartzler R. U. (2016). Arthroscopic Superior Capsular Reconstruction for Massive Irreparable Rotator Cuff Repair. Arthrosc. Tech. 5, e1407–e1418. 10.1016/j.eats.2016.08.024 28149739 PMC5264010

[B16] Chouhan D. Mandal B. B. (2020). Silk Biomaterials in Wound Healing and Skin Regeneration Therapeutics: From Bench to Bedside. Acta Biomater. 103, 24–51. 10.1016/j.actbio.2019.11.050 31805409

[B17] Clark R. A. F. (1993). Regulation of Fibroplasia in Cutaneous Wound Repair. Am. J. Med. Sci. 306, 42–48. 10.1097/00000441-199307000-00011 8328509

[B18] Denard P. J. Brady P. C. Adams C. R. Tokish J. M. Burkhart S. S. (2018). Preliminary Results of Arthroscopic Superior Capsule Reconstruction with Dermal Allograft. Arthrosc. J. Arthrosc. Relat. Surg. 34, 93–99. 10.1016/j.arthro.2017.08.265 29146165

[B19] Ferrando A. Kingston R. Delaney R. A. (2021). Superior Capsular Reconstruction Using a Porcine Dermal Xenograft for Irreparable Rotator Cuff Tears: Outcomes at Minimum Two-Year Follow-Up. J. Shoulder Elb. Surg. 30, 1053–1059. 10.1016/j.jse.2020.08.020 32890682

[B20] Flury M. Rickenbacher D. Jung C. Schneider M. M. Endell D. Audigé L. (2018). Porcine Dermis Patch Augmentation of Supraspinatus Tendon Repairs: A Pilot Study Assessing Tendon Integrity and Shoulder Function 2 Years after Arthroscopic Repair in Patients Aged 60 Years or Older. Arthrosc. J. Arthrosc. Relat. Surg. 34, 24–37. 10.1016/j.arthro.2017.06.024 28822637

[B21] Greenhalgh D. G. (1998). The Role of Apoptosis in Wound Healing. Int. J. Biochem. Cell. Biol. 30, 1019–1030. 10.1016/S1357-2725(98)00058-2 9785465

[B22] Kalem M. (2015). Role of Anti-adhesive Barriers Following Rotator Cuff Repair Surgery: an Experimental Study. Acta Orthop. Traumatol. Turc. 50 (2), 227–233. 10.3944/AOTT.2015.15.0134 26969960

[B23] Kalina R. Neoral P. Holibka R. Gallo J. (2019). Arthroscopic Superior Capsule Reconstruction Using the DX Reinforcement Matrix in Patients with Irreparable Rotator Cuff Tears - Pilot Data. Acta Chir. Orthop. Traumatol. Cech 86, 264–270. 31524587

[B24] Mihata T. Lee T. Q. Watanabe C. Fukunishi K. Ohue M. Tsujimura T. (2013). Clinical Results of Arthroscopic Superior Capsule Reconstruction for Irreparable Rotator Cuff Tears. Arthrosc. J. Arthrosc. Relat. Surg. 29, 459–470. 10.1016/j.arthro.2012.10.022 23369443

[B25] Mihata T. McGarry M. H. Pirolo J. M. Kinoshita M. Lee T. Q. (2012). Superior Capsule Reconstruction to Restore Superior Stability in Irreparable Rotator Cuff Tears. Am. J. Sports Med. 40, 2248–2255. 10.1177/0363546512456195 22886689

[B26] Mir M. Ali M. N. Barakullah A. Gulzar A. Arshad M. Fatima S. (2018). Synthetic Polymeric Biomaterials for Wound Healing: a Review. Prog. Biomater. 7, 1–21. 10.1007/s40204-018-0083-4 29446015 PMC5823812

[B27] Nauta A. Gurtner G. Longaker M. (2011). Wound Healing and Regenerative Strategies. Oral Dis. 17, 541–549. 10.1111/j.1601-0825.2011.01787.x 21332599

[B28] Ono Y. Dávalos Herrera D. A. Woodmass J. M. Boorman R. S. Thornton G. M. Lo I. K. Y. (2016). Healing Rate and Clinical Outcomes of Xenografts Appear to Be Inferior when Compared to Other Graft Material in Rotator Cuff Repair: a Meta-Analysis. J. ISAKOS 1, 321–328. 10.1136/jisakos-2016-000076

[B29] Perl A. (2017). Review: Metabolic Control of Immune System Activation in Rheumatic Diseases. Arthritis Rheumatol. 69, 2259–2270. 10.1002/art.40223 28841779 PMC5711528

[B30] Polacek M. (2019). Arthroscopic Superior Capsular Reconstruction with Acellular Porcine Dermal Xenograft for the Treatment of Massive Irreparable Rotator Cuff Tears. Arthrosc. Sports Med. Rehabilitation 1, e75–e84. 10.1016/j.asmr.2019.08.001 PMC7120814 32266343

[B31] Ravenscroft M. J. Riley J. A. Morgan B. W. Sandher D. S. Odak S. S. Joseph P. (2019). Histological Incorporation of Acellular Dermal Matrix in the Failed Superior Capsule Reconstruction of the Shoulder. J. Exp. Ortop. 6, 21. 10.1186/s40634-019-0189-1 PMC6535153 31129749

[B32] Sher J. S. Uribe J. W. Posada A. Murphy B. J. Zlatkin M. B. (1995). Abnormal Findings on Magnetic Resonance Images of Asymptomatic Shoulders. J. Bone & Jt. Surg. 77, 10–15. 10.2106/00004623-199501000-00002 7822341

[B33] Spolarics Z. Navarro L. (1994). Endotoxin Stimulates the Expression of Glucose-6-Phosphate Dehydrogenase in Kupffer and Hepatic Endothelial Cells. J. Leukoc. Biol. 56, 453–457. 10.1002/jlb.56.4.453 7930940

[B34] Taylor S. H. Al-Youha S. van Agtmael T. Lu Y. Wong J. McGrouther D. A. (2011). Tendon Is Covered by a Basement Membrane Epithelium that Is Required for Cell Retention and the Prevention of Adhesion Formation. PLoS ONE 6, e16337. 10.1371/journal.pone.0016337 21298098 PMC3027644

[B35] Tempelhof S. Rupp S. Seil R. (1999). Age-related Prevalence of Rotator Cuff Tears in Asymptomatic Shoulders. J. Shoulder Elb. Surg. 8, 296–299. 10.1016/S1058-2746(99)90148-9 10471998

[B36] Toolan B. C. (2006). Current Concepts Review: Orthobiologics. Foot Ankle Int. 27, 561–566. 10.1177/107110070602700715 16842727

[B37] Xue M. Jackson C. J. (2015). Extracellular Matrix Reorganization during Wound Healing and its Impact on Abnormal Scarring. Adv. Wound Care 4, 119–136. 10.1089/wound.2013.0485 PMC4352699 25785236

[B38] Yamaguchi K. Tetro A. M. Blam O. Evanoff B. A. Teefey S. A. Middleton W. D. (2001). Natural History of Asymptomatic Rotator Cuff Tears: A Longitudinal Analysis of Asymptomatic Tears Detected Sonographically. J. Shoulder Elb. Surg. 10, 199–203. 10.1067/mse.2001.113086 11408898

[B39] Yildiz F. Bilsel K. Pulatkan A. Kapicioglu M. Uzer G. Çetindamar T. (2019). Comparison of Two Different Superior Capsule Reconstruction Methods in the Treatment of Chronic Irreparable Rotator Cuff Tears: a Biomechanical and Histologic Study in Rabbit Models. J. Shoulder Elb. Surg. 28, 530–538. 10.1016/j.jse.2018.08.022 30466819

[B40] Zarei F. Soleimaninejad M. (2018). Role of Growth Factors and Biomaterials in Wound Healing. Artif. Cells, Nanomedicine, Biotechnol. 46, 906–911. 10.1080/21691401.2018.1439836 29448839

[B41] Zhao S. Su W. Shah V. Hobson D. Yildirimer L. Yeung K. W. K. (2017). Biomaterials Based Strategies for Rotator Cuff Repair. Colloids Surfaces B Biointerfaces 157, 407–416. 10.1016/j.colsurfb.2017.06.004 28633121

