# Supplementary figures and images for: Evaluation of the Cell Behavior and Growth Characteristics of the Porcine Dermal Xenograft Patch in Relation to the Surface Properties
Source: Front Bioeng Biotechnol. 2022 May 30;10:811446. doi: 10.3389/fbioe.2022.811446 (PMC9189373; doi:10.3389/fbioe.2022.811446)

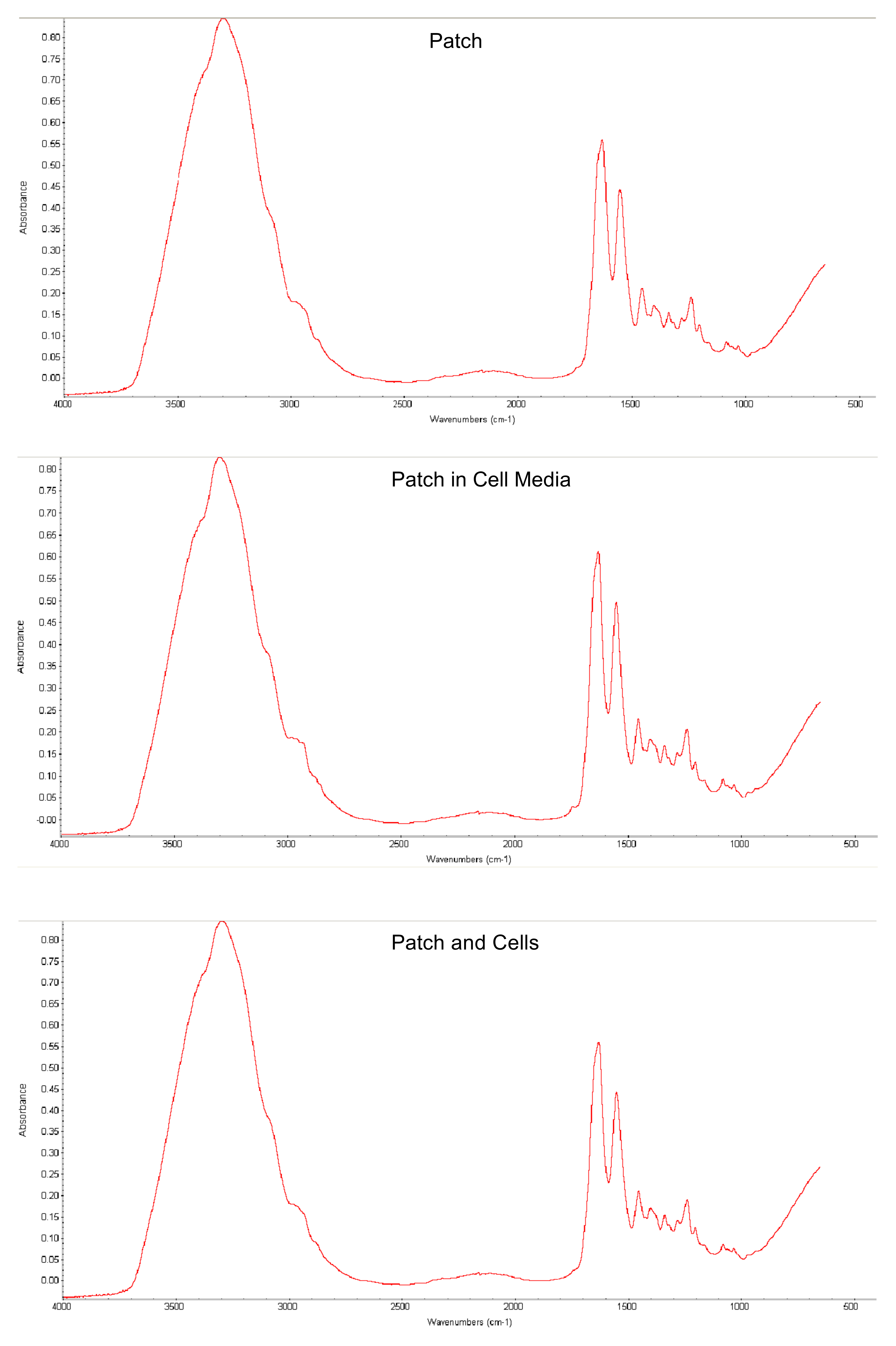

Supplement: Supplementary file 1 [file Image1.TIF]
